# Analytical Quality by Design Approach of Reverse-Phase High-Performance Liquid Chromatography of Atorvastatin: Method Development, Optimization, Validation, and the Stability-Indicated Method

**DOI:** 10.1155/2021/8833900

**Published:** 2021-02-13

**Authors:** Nabil K. Alruwaili

**Affiliations:** Department of Pharmaceutics, College of Pharmacy, Jouf University, Aljouf, Sakakah, Saudi Arabia

## Abstract

The use of analytical quality by design (AQbD) approach in the optimization of the high-performance liquid chromatography (RP-HPLC) method is a novel tool. Three factors and three levels of Box–Behnken statistical design (BBD) were used for method optimization and analysis of atorvastatin. The mobile phase (acetonitrile: water), flow rate (Rt), and UV wavelength were used as independent variables. Their effects were observed in the area of the chromatogram (AU), retention time (Rt, min), and tailing factor (%). The optimized HPLC condition was found as acetonitrile:water (50 : 50), flow rate (0.68 ml/min), and UV wave length (235 nm). It gives the retention time of 2.43 min with the linearity range of 5–30 *μ*g/ml with a high regression value (*r*^2^ = 0.999). The method was found to be precise and accurate with low % RSD (<5%). The refrigeration stability indicated that atorvastatin was stable. The force degradation study showed that the atorvastatin was fully unstable in UV light and stable in 0.1 M basic condition. It concluded that this QbD optimized method is suitable for quantification of the atorvastatin from the formulation as well as pharmacokinetic parameters.

## 1. Introduction

**Figure 1:**
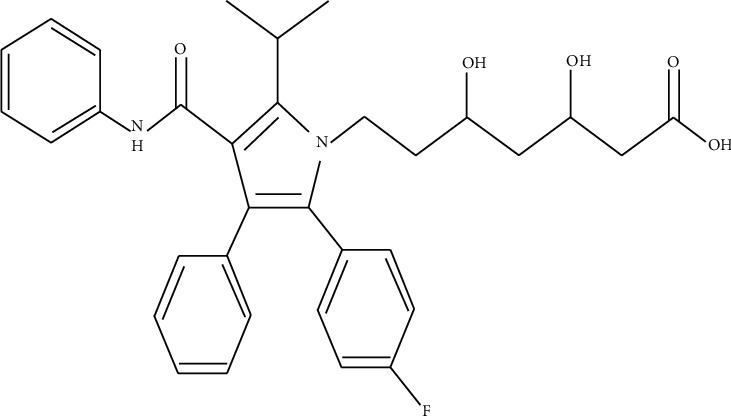
Chemical structure of atorvastatin.

Atorvastatin (ATS) belongs to statin class and is used for lowering the lipid in the body through desensitizing the making of cholesterol in the liver resulting in decreasing the risk of cardiovascular diseases. Mainly, it completely inhibited hydroxymethylglutaryl coenzyme A reductase enzyme [1, 2]. Chemically, it is (3R, 5R)-7-[2-(4-flurophenyl)-3-phenyl-4-(phenylcarbamoyl)-5-propane-2-ylpyrrol-1-yl]-3,5 dihydroxy eptanoic acid [3] (Figure 1). ATS is insoluble in water and soluble in acetonitrile, methanol, ethanol, dimethyl sulfoxide, and other organic solvents [4, 5].

The RP-HPLC is a very sensitive analytical technique for estimation of the drug from the formulation as well as a biological sample [6]. Some HPLC methods for ATS literature have been reported earlier by a researcher, i.e., simultaneous estimation of ATS, amlodipine, and benazepril [7, 8]. Erturk et al. reported the HPLC method for estimation of ATS from the bulk drug and tablet [9, 10]. Martins and associates estimated the ATS from the biological sample [11], but ATS did not well resolve and has longer retention time (Rt).

Several published studies reported the relevance of quality by design approach for optimization of the HPLC method and provide prized information about the interaction effect of the variable over the response as well as give appropriated chromatographic condition in which drug is well separated [12, 13]. The analytical quality by design (AQbD) is a novel tool for optimization of HPLC instrumental conditions such as flow rate, the solvent system, solvent ratio, the effect of temperature, injection volume, the resolution of the drug (Rt) as well as cost and effort. The AQbD gives information on risk associated with the method as well as the influencing factor effect on analytical method performance. Various research publications have been explained by the application of AQbD approaches [14–16]. This approach can create various analytical chromatographic conditions with noticeably improved method presentation. This approach also helps in determining the crucial method variables, which directly impact the performance, enlarge the least resources of time, endeavour, and cost. It helps to understand the risk assessment of the critical factors which are directly influenced in the presentation of the analytical method [17, 18]. It is based on the principles of experimental design that participated in a systematic thoughtful of the reasonable risk and interaction between the experimental variables [19]. The Box–Behnken design has various advantages such as prevent the waste of solvent and time. It gives a lesser number of runs in the various compositions of independent variables. There are many published research works on optimization of RP-HPLC using QbD (Box–Behnken design) reported such as voriconazole [20], methotrexate [21], dapagliflozin [12], lacidipine [13], timolol maleate [22], risperidone [23], and temozolomide [24]. No method has been reported on the RP-HPLC method using a QbD approach for atorvastatin. This research work developed and optimized the analytical condition by using AQbD (Box–Behnken design).

## 2. Materials and Methods

### 2.1. Materials

Atorvastatin (ATS, 99.99%) as a gift sample was obtained from Aldrich (St. Louis, US). HPLC grade water, methanol, and acetonitrile were procured from Sigma Aldrich (St. Louis, US). All other chemicals used in this study are analytical grade.

### 2.2. Methods

#### 2.2.1. Liquid Chromatographic Condition

High-performance liquid chromatography (HPLC, Auto sampler, Shimadzu LC10AD, Kyoto Japan) attached with a UV detector (SPD-20A) was used for the method development of ATS. The ultrafine particle packed 250 mm length C_18_ column (Acclaim120C18, 4.6 mm internal diameter, 2.2 *μ*m particle size) was used for chromatographic separation of ATS. Flow range, i.e., 0.1–1 ml/min, was used for separation of ATS. The different mobile phase compositions of acetonitrile and water (50 : 50, 60 : 40, and 70 : 30) were used as mobile phase. The whole operation was carried out at room temperature. The sample (30 *μ*L) was applied as injection volume for each sample.

#### 2.2.2. Risk Assessment Study

This study was conducted to understand the various factors affecting the target quality profile (TQP). The critical analytical attributes were applied to visualize the proper relationships between parameters to the target quality profile before risk judgment. This study was used to classify the possible causes of difficulty to obtain the reason for deficiency, discrepancy, and failure (defect). It is also applied to determine the considerable role for the extraction of vital detail required to achieve the target quality profile. The factors which influence the target response are categorized as a low, medium, and high score [25]. The seven factors were used and further divided into low, medium, and high-risk levels for screening and obtaining only a few factors. Finally, three out of seven factors were selected for the optimization of responses using experiment designs (Table 1). Using this study, the target quality profile was recognized in the form of retention time (Rt), area, and tailing factor from the independent seven variables.

#### 2.2.3. Optimization

The AQbD method was used to analyze the effect of independent factors onto the responses. Box–Behnken design (BBD, Design Expert 8.0.7.1, Stat-Ease Inc., Write country) is used for the optimization because it gives a few numbers of experimental trials with appropriate composition [21]. The three-factor and three-level BBD were used for the optimization of an analytical method. The mobile phases, i.e., acetonitrile (X_1_, %), flow rate (X_2_, ml/min), and UV wavelength (X_3_) and their single and combined effects were examined over the selected responses, i.e., area (Y_1_), Rt (min, Y_2_), and tailing factor (%, Y_3_). In total, 17 experimental runs at various compositions were obtained from software to find out the best composition (Table 2). The experimental data were fitted into different kinetic models such as linear, second-order, quadratic, and cubic models to find the best fit model. The polynomial equation, 3D plot, and contour plot were measured to evaluate the effect of the variable on responses. Statistical analysis ANOVA and regression analysis were performed to determine the significant level for the model and to determine the best fit model.

#### 2.2.4. Preparation of Standard Solution and Linearity Curve

The standard solution was prepared by weighing ATS (10 mg) and dissolved in HPLC grade methanol (10 mL). From this stock solution, further dilution was performed to prepare the concentration range of 5–30 *μ*g/ml using methanol. Each sample was injected (20 *μ*L) in the HPLC instrument by an autosampler, and the different parameters were determined. The linearity curve was plotted between the area of chromatogram vs. concentration (*μ*g/ml), and the linear regression equation was calculated. The analysis was performed in triplicate, and the mean value with standard deviation was used.

#### 2.2.5. Quality Control Sample

For validation of the method, the quality control sample was prepared to form a stock solution at three levels, i.e., low-quality control (1.5 *μ*g/ml), the medium limit of quality control (MQC 12.43 *μ*g/ml), and high-quality control (HQC, 24 *μ*g/ml).

#### 2.2.6. Method Validation

HPLC method was validated according to the approved protocol given in ICH Q2 (R1). The method was validated for linearity, accuracy, sensitivity, precision, recovery, LOD, and LQC of the quality control sample [26, 27].

#### 2.2.7. System Suitability and Specificity

The system suitability of the method was performed, and the area, Rt, and tailing factor were assessed with the same concentration of ATS injection in six replicates. The specificity of the method was assessed to find out any interference on chromatographic separation of the ATS with a blank sample.

#### 2.2.8. Linearity and Sensitivity Analysis

The linearity study of ATS was analyzed at a concentration range of 5–30 *μ*g/ml. The prepared sample was injected into the HPLC instrument, and the peak area was noted. The linearity curve was plotted between AST concentrating vs. the peak area. The slope and regression coefficient were calculated from the curve. The study was carried out in triplicate. The limit of detection (LOD) and limit of quantification (LOQ) were analyzed by signal-to-noise ratio of 3 : 1 and 10 : 1 level and calculated by the following formula.

(1) $$\begin{array}{c} \text{LOD}=3.3\times \frac{\text{Standard deviation }}{\text{Slope}},\\ \text{LOQ}=10\times \frac{\text{Standard deviation }}{\text{Slope}}. \end{array}$$

#### 2.2.9. Precision and Accuracy

Accuracy and precision studies were performed to determine the closeness between several injections of the same concentration. The study was conducted several times on the same day and on different days. The intraday (within a day) and interday (different days) precision and accuracy of the method were analyzed at three different quality samples, i.e., LOC (1.5 *μ*g/ml), MQC (12.43 *μ*g/ml), and HQC (24 *μ*g/ml) for six replicates three times a day and three different days. The regressed standard deviation (% RSD) was calculated for each study.

#### 2.2.10. Recovery Studies

The recovery study of ATS was estimated by spiking the sample after the addition of an extra quantity of standard ATS (50, 100, and 150%). The analysis was performed by HPLC using an optimized mobile phase, flow rate, and UV wavelength in triplicate. The % recovery was calculated by the given formula:

(2) $$\begin{array}{c} \%\text{ Recovery}=\frac{\text{Recovered concentration}}{\text{Initial concentration}}\times 100. \end{array}$$

#### 2.2.11. Storage Stability Study

The refrigerated stability of ATS was analyzed at LQC and HQC concentration. The study was conducted at different temperatures for long-term, bench-top, freeze-thaw, and postprocessing stability. The long-term ATS stability was performed at −80°C for 30 days in the deep freezer. The freeze-thaw ATS stability was performed at −20°C to 25°C for three successive days. The bench-top study was carried out at 25°C after 24 h of storage. The postoperation study was carried out at 10°C by storing the sample in an autosampler of HPLC. The study was conducted in six replicates, and drug concentration as well as % RSD was calculated.

#### 2.2.12. Forced Degradation Study

Stress degradation study provides information about the stability of the drug and drug product. It gives information about the stability of substances with acid, base, light, pH, hydrolysis, and oxidation condition. It directly affects the selection of formulation development, packaging, storage, transportation, shelf life, chemical stability of the drug, and drug product [28, 29].

#### 2.2.13. Acid and Base Degradation Analysis

The study was conducted with 1 M hydrochloric acid and 0.1 M sodium hydroxide. An equal volume ratio of the ATS stock solution was taken and added to acid and base solutions and mixed into the round bottom flask. The mixture was refluxed using an air reflux condenser at 90°C for 6 h, and then, the sample was cooled at room temperature. The sample was appropriately diluted with HPLC methanol, filtered through with the membrane filter (0.45 *μ*m), and injected into the HPLC instrument.

#### 2.2.14. Oxidation Degradation Study

The oxidation degradation study was carried out by using the oxidizing agent hydrogen peroxide (30%). The standard stock solution of ATS was mixed with hydrogen peroxide in equal volume. The sample was refluxed with air condenser at 90°C for 6 h and cooled at room temperature, diluted with methanol. The sample (20 *μ*l) was injected into the HPLC instrument and analyzed for drug content.

#### 2.2.15. Photolytic Degradation Study

The photodegradation study was conducted to evaluate the effect of UV light during the processing of API for formulation development. For this study, the standard stock solution of ATS placed into an open quartz glass vial and exposed with UV light (200 watt/square meter) for 24 h. Then, the sample was appropriately diluted with HPLC grade methanol and analyzed by HPLC.

## 3. Result and Discussion

### 3.1. Method Development

The RP-HPLC analytical method of ATS was developed and optimized by AQbD. The various mobile phases such as methanol, acetonitrile, water, and phosphate buffer in different compositions were used with different flow rates to obtain a suitable RP-HPLC method. Among different mobile systems with different ratios, a well-resolved peak was not found. The retention time was also found to be high. Finally, acetonitrile and water as the mobile phase was tried at 50 : 50 v/v and got a well-determined peak at 2.43 min using a flow rate of 0.5 ml/min, at 235 nm. The further mobile phase, flow rate, and UV wavelength were used to optimize BBD software for a robust method.

### 3.2. Risk Assessment Study

The risk assessment study was carried out for the determination of essential constraint of the method and to get the robust condition. It shows the connection between various parameters and analytical attributes. As per the examination of previously published research work descriptions, various possible input parameters were measured at a different level of risks connected with each experimental parameter of the HPLC method, i.e., the ratio of mobile phase and flow rate. It was found that wavelength and flow rate in the medium risk zone and other constraints have less risk due to the negligible effect on the developed method. There is continuous improvement in the design space that has been found with the use of risk management and screening, experimental design, and response surface methodology to get an optimum analytical condition. The use of the high, medium, and low-risk factors is depicted in Table 1.

### 3.3. Optimization

**Figure 2:**
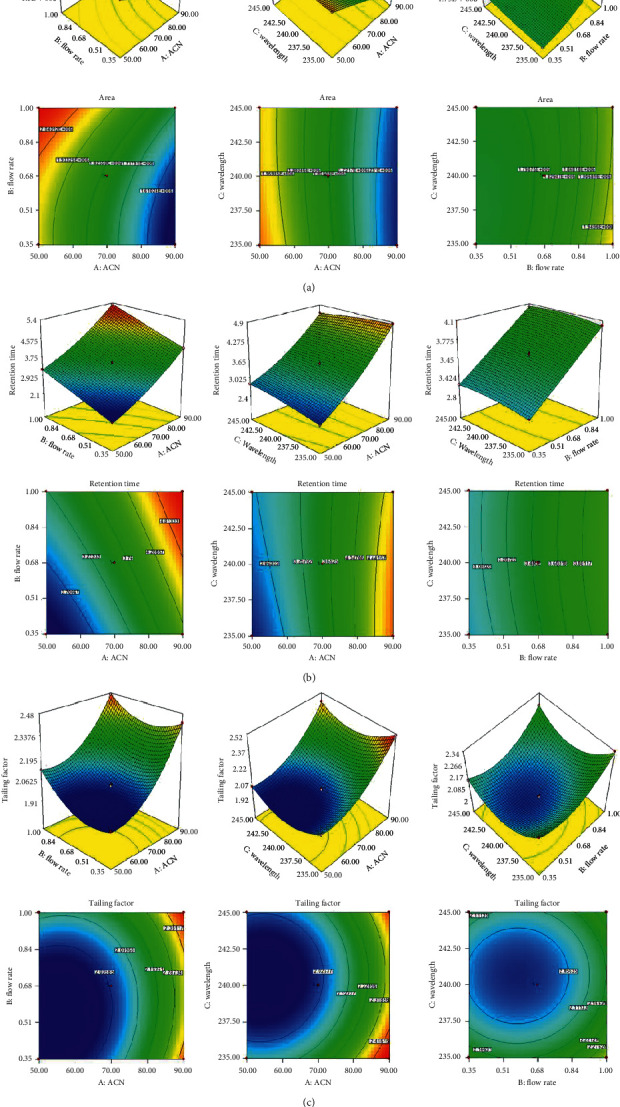
(a) Effect of acetonitrile, flow rate, and wavelength on the area. (b) Effect of acetonitrile, flow rate, and wavelength on the retention time. (c) Effect of acetonitrile, flow rate, and wavelength on the tailing factor.

The BBD software was used for the optimization of analytical (HPLC) parameters to get the optimum conditions for the estimation of ATS. The independent variables such as acetonitrile (50–90% v/v), flow rate (0.35–1 ml/min), and wavelength (235–245 nm) were used to determine the effect on dependent variables (Rt, area, and tailing factor). Total seventeen experimental runs with five centre points obtained from the software for determination of optimum chromatographic separation of ATS (Table 2). The variables, i.e., mobile phase ratio (water: acetonitrile 30 : 70% v/v), flow rate (0.7 ml/min), and wavelength (240 nm), are the centre point of composition. The desirable factor was found to be very close to one (0.9653) at centre point, indicating well-fitting of the model, and the responses were within the target value. The effects of variables over the responses were evaluated to select the design space. The data of responses were fitted into different polynomial designing models such as linear, second-order, quadratic, and cubic. The quadratic model was found to be the best fit model because its regression coefficient (*R*^2^ = 0.9999) is significantly higher (*p* < 0.0001) than other models. The ANOVA of each model was evaluated, and parameters such as the sum of the square, *F* value, mean square, and *p* value of each response were also calculated. The 3D and contour plot of each response was constructed from the software and demonstrated the effect of multiple variables (integration effect) on a response at one time (Figures 2(a)–2(c)).

### 3.4. Effect of Independent Factors on Peak Area

The effect of an independent variable on the peak area was expressed by the 3D response, contour plot, and polynomial equation. The 2^nd^ order quadratic polynomial for response *Y*_1_ (area) is given as follows.

Area (*Y*_1_) = 1804782–222698.48*X*_1_ − 100301.57*X*_2_ − 2901.74*X*_3_ + 13807.15 *X* _1_ *X* _2_ +14093.63*X*_1_*X*_3_ − 14694.06 *X*_2_*X*_3_ − 16298.40 *X*_1_^2^ + 50901.77 *X*_2_^2^ + 10629.64 *X*_3_^2^.

In this polynomial equation, the *X*_1_, *X*_2_, *X*_3_, *X*_1_*X*_2_, *X*_1_*X*_3_, *X*_2_*X*_3_, *X*_1_^2^, *X*_2_^2^, and *X*_3_^2^ are the significant model terms because all the model terms have *p* < 0.05. The *F* value of the quadratic model was found to be 32195.28, and it implies that the model is significant. The predicted *R*^2^ (0.9996) is closer to the adjusted *R*^2^ (0.9999). The adequate precession is greater than four (646.328), and it indicated that the model has sufficient signal. The polynomial equation showed that acetonitrile (*X*_1_, ACN) has shown a positive effect, whereas flow rate (*X*_2_) and wavelength (*X*_3_) have a negative effect on the peak area. The peak area linearly decreases with an increase in the acetonitrile percentage with respect to water, flow rate, and wavelength (Figure 2(a)). But the percentage of acetonitrile and flow rate has a dominant effect on the peak area of chromatogram as compared to detector wavelength.

### 3.5. Effect of Independent Factors on Retention Time

The effect of an independent variable on the retention time was expressed by the 3D response, contour plot, and polynomial equation. The 2^nd^ order quadratic polynomial equation for the response *Y*_2,_ retention time, is given as follows.

Retention time (*Y*_2_) = +3.54 + 1.02*X*_1_ + 0.55*X*_2_ + 0.024*X*_3_ + 0.027*X*_1_*X*_2_ − 0.1*X*_1_*X*_3_ − 0.015 *X*_2_*X*_3_ + 0.21 *X*_1_^2^ − 0.018 *X*_1_^2^ − 0.037*X*_1_^2^.

The effects of the different variables on flow rate were expressed by the polynomial equation, 3D plot, and contour plots. The factors *X*_1_, *X*_2_, and *X*_3_ showed the synergistic effect on the response. On increasing the % of acetonitrile (*X*_1_), the Rt of ATS increased. The high increment of Rt is not good for analysis because of the wastage of the solvent as well as time. So, it selected the medium percentage of solvent for analysis. The flow rate (*X*_2_) increases as the Rt value increases slightly as compared to acetonitrile. The design showed the quadratic model as the best fit model because the regression coefficient is closer to one (*R*^2^ = 0.9999) than other analytical experimental design models. The model *F* value (5910.01) implies that the model is significant (*p* < 0.05). All the model terms (*X*_1_, *X*_2_, *X*_3_, X_1_*X*_2_, X_1_*X*_3_, *X*_2_*X*_3_, *X*_1_^2^, *X*_2_^2^, and *X*_3_^2^) have *p* < 0.05, indicating a significant model term. The predicted *R*^2^ (0.9997) is closer to the adjusted *R*^2^ (0.9979). The adequate precession value from the software represented is greater than four (1923.940) and indicates that the model has sufficient signal. The 3D and contour plot showed that the Rt increases with increase in the flow rate (Figure 2(b)). In this method, the medium level of flow rate (ml/min) was selected as an optimized condition for the method development. The third-factor wavelength showed increases in the UV wavelength; the Rt value of ATS slightly increases as compared to other factors, i.e., acetonitrile and flow rate. The interaction effect (combined) of acetonitrile, wavelength (*X*_1_*X*_3_) and flow rate, wavelength (*X*_2_*X*_3_) showed an antagonistic effect on the Rt of ATS.

### 3.6. Effect of Independent Factors on Tailing Factor

The effect of an independent variable on the retention time was expressed by the 3D response, contour plot, and polynomial equation. The 2^nd^ order quadratic polynomial equation for the response *Y*_3_ (tailing factor) is given as follows.

Tailing factor (*Y*_3_) = +2.012 + 0.206*X*_1_ + 0.059*X*_2_  − 0.032*X*_3_  −  0.03*X*_1_*X*_2_  −  0.008*X*_1_*X*_3_  −  0.002_3_ +  0.129*X*_1_^2^ +  0.099*X*_1_^2^ + 0.127*X*_3_^2^.

The quadratic model was found to be the best model rather than other analytical experimental designing models. The regression coefficient is found close to one (*R*^2^ = 0.9993) (Table 3). The model terms *X*_1_, *X*_2_, *X*_2_, X_1_*X*_2_, *X*_1_^2^, *X*_2_^2^, and *X*_3_^2^ are the significant effects on response (*p* < 0.05) and other *X*_1_*X*_3_ and *X*_2_*X*_3_ are insignificant (*p* > 0.1) on response. The model *F* value was found too high (1113.03) and implies that the model is significant. The *F* value of lack fit was found to be less (5.42), and it indicates that the model is nonsignificant (*p* > 0.1); it is good for the fitted quadratic model. The variable effect on the tailing factor was evaluated by the 3D surface and contour plot. It showed the mobile phase system and flow rate favoured the response, whereas detector UV wavelength has a negative response. The 3D and contour plot showed that increasing acetonitrile percentage (*X*_1_) increases the tailing factor (Figure 2(c)). The high and lesser values of the tailing factor are not suggested for the analytical method (HPLC). For this selected medium, acetonitrile concentration was found to be within the standard limit [30]. The 2^nd^ factor solvent flow rate (*X*_2_) increases; the tailing factor also increases but has a less prominent effect than factor A (Figure 2(c)). Moreover, the third-factor UV wavelength (*X*_3_) has a negative effect on the tailing factor, which means UV wavelength increases from 235 nm to 245 nm and the tailing factor decrease. The interaction effect of acetonitrile (A) and flow rate showed the negative effect on the tailing factor, and other interaction factors (*X*_2_*X*_3_ and *X*_1_*X*_3_) showed no significant effect (*p* > 0.1) on the tailing factor.

### 3.7. Optimization by Point Prediction

The further optimization of the chromatographic condition was carried out by point prediction. The changes in the mobile phase composition, flow rate, and wavelength were carried out in the software. The optimized chromatographic condition, i.e., mobile phase (50 : 50%), wavelength 235 nm, and flow rate 0.7 ml/min, shows the responses for area as 2036620 AU, retention time 2.43 min, and tailing 2.08%. It showed less error (%) from the predicted value (software value).

## 4. Validation

### 4.1. System Suitability and Specificity

**Figure 3:**
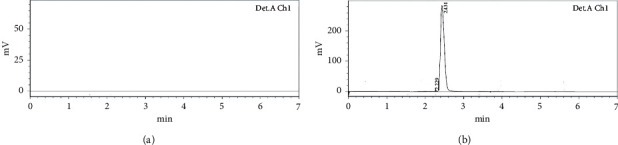
HPLC chromatogram of (a) blank sample and (b) atorvastatin sample.

The system suitability of ATS was determined at different responses or critical analytical attributes, i.e., the area of the chromatogram, Rt, and tailing effect. It was found that there are no significant (*p* > 0.05) differences in responses after the evaluation of six replicates of analysis. The RSD (%) system suitability of the method found to be <1 (0.73) represented the high scale of precision of instrument [31]. The specificity of the developed analytical chromatographic method was performed in the blank and spiked quality control samples, and it was found that there is no interference with blank and standard quality control samples (Figures 3(a) and 3(b)). It confirmed that the method was suitable and specific for the quantification of ATS from the formulation.

### 4.2. Linearity and Sensitivity Analysis

The standard curve of ATS was conducted at 5–30 *μ*g/ml standard solution using optimized chromatographic conditions (Table 4). The regression coefficient (*R*^2^) was found to be 0.999, and the regression equation was 98881*x* + 71.42. The RSD of slope and intercept was calculated and found as 0.89% and 0.76%, respectively. It was found as less than 5%, and it indicates that the method was good and accurate. The limit of detection and limit of quantification was found to be 0.25 *μ*g/ml and 0.83 *μ*g/ml, respectively.

### 4.3. Precision and Accuracy

The precision and accuracy of the developed method at an optimized chromatographic condition in different quality control samples were analyzed in six replicates, and the data are expressed in Table 5. The percentage RSD was determined and found between 1.70 and 2.79 for intraday and 1.25 and 2.71 for interday at LQC, MQC, and HQC quality control sample. RSD was <5%, and as per the guidelines, it indicates that the method was accurate and suitable [32, 33]. The percentage accuracy of the quality control sample (LQC, MQC, and HQC) was found to be 98.66 ± 2.86–99.83 ± 1.79 for intraday and 98.33 ± 2.46–99.91 ± 2.33 for interday, respectively. The accuracy of the method was found to be within the acceptable limit of the guideline.

### 4.4. Recovery Study

The recovery study of ATS was determined by adding an extra amount of drug into analytes. The recovery of ATS was found to be 98.66–100.26, respectively, with the low RSD (0.98–1.91%). The low RSD (<5%) indicates that the method was accurate.

### 4.5. Storage Stability Study

The refrigerated storage stability of ATS was analyzed at two levels of quality samples, i.e., LQC and HQC (1.5 *μ*g/mL and 24 *μ*g/mL), and the result is given in Table 6. The percentage recovery at the LQC level was 99.33–100% and at the HQC level was 99.58–100% in different storage conditions, i.e., short-term stability (25°C, 24 h), long-term stability (−80°C, 30 days), freeze-thaw stability (three cycles, −20°C to 25 C), postpreparative stability (10°C, 24 h), and short-term stability (25°C, 24 h). There was no significant (*p* > 0.05) change found with a low value of % RSD (<5%). The low value of RSD indicated that ATS was stable at different freezing storage conditions.

## 5. Forced Degradation Study

### 5.1. Acid Degradation Study

**Figure 4:**
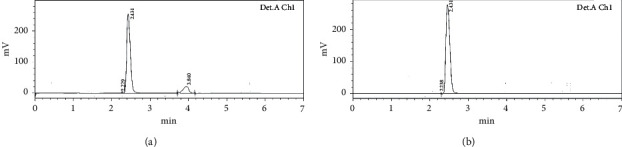
HPLC chromatogram of atorvastatin: (a) acid degradation and (b) base degradation.

The chromatogram of ATS after the degradation study showed one additional peak at 3.8 min except than the standard ATS peak (2.43 min) (Figure 4(a)). The additional peak indicated that ATS was unstable at a high molar concentration of HCl as well as at high temperature. As per previous work' report, the most prominent acid degradation product is lacton. The degradation product form by lactonization of 3,5-dihydroxyheptanoate side chain under mild acidic condition and further may degrade upon exposure with strong acid for long exposure time [34, 35].

### 5.2. Basic Degradation Study

The base degradation study was conducted in 0.1 M sodium hydroxide, and no additional peaks were found in the chromatogram of AST (Figure 4(b)). There was 100% concentration retained, and it indicates that the drug was stable in basic condition.

### 5.3. Oxidative Degradation Study

**Figure 5:**
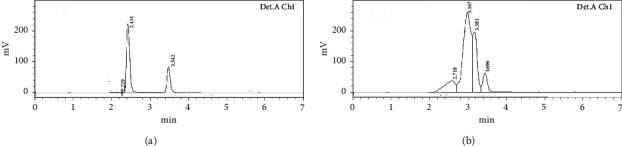
HPLC chromatogram of atorvastatin: (a) oxidative degradation and (b) UV light degradation.

An oxidation degradation study was conducted using hydrogen peroxide solution (30% v/v) under reflex condition. One additional well-resolved peak was observed at Rt 3.54 min in addition to the standard ATS peak (Rt = 2.43 min) (Figure 5(a)). The mechanism of hydrogen peroxide oxidation takes place by oxidation of pyrrole ring of ATS and may be the formation of unstable endoperoxide that takes place [36].

### 5.4. UV Photolytic Degradation Study

The study was carried out by placing the standard sample under UV light (254 nm) for 24 h, and chromatogram is depicted in Figure 5(b). There are many ATS peaks observed in the degradation chromatogram of ATS. The degradation peaks were observed at different Rts (2.71 min, 3.16 min, 3.38 min, and 3.7 min). It indicates that ATS was completely degraded under UV light after long time exposure due to absorption of energy from UV light by molecules. The molecule is absorbed and indulgences the energy in presence of UV light. ATS molecule activated from the ground to excited state because it contains the carbonyl and *c* = *c* group as well as the formation of free radical takes place [37].

## 6. Conclusion

An analytical quality by design (Box–Behnken) approach was successfully employed for the optimization of the RP-HPLC method. The solvent system (acetonitrile:water, 50 : 50), flow rate (0.7 ml/min), and UV wavelength (235 nm) were found to be the optimized chromatographic condition for atorvastatin. The developed method was evaluated for the validation, quantification of the quality control sample, and stability study. The method showed a well-resolved chromatographic peak (Rt = 2.43 min) and found to be accurate and precise. The force degradation study showed well-resolved atorvastatin and degradation product peaks. Our finding suggests that QbD approaches can be used for the method development and also for the determination of atorvastatin from formulation and pharmacokinetic parameter in vivo.

## Figures and Tables

**Table 1:** RP-HPLC analytical method risk assessment for atorvastatin.

Critical attributes response	Various RP-HPLC chromatographic conditions for atorvastatin							
	Composition of mobile phase	UV wavelength	Injection volume	Column temperature	Flow rate	Sample temperature	Column dimension	Type of vial
Chromatogram area	+2	+1	−1	−1	+2	−1	+1	−1
Retention time	+2	+1	−1	+1	+2	−1	+1	−1
Tailing factor	+2	+1	−1	−1	+1	−1	+1	−1

+2, high-risk parameter; +1, medium-risk parameter; −1, low-risk parameter.

**Table 2:** Critical influencing variable and their actual and predicted values of responses.

Standard order	Critical influencing variable			Response					
	Acetonitrile (%)	Flow rate (ml/min)	Wavelength (nm)	Area (AU)		Retention time		Tailing factor	
				Actual value	Predicted value	Actual value	Predicted value	Actual value	Predicted value
1	50	0.35	240	1975990	1975589	2.19	2.18	1.95	1.94
2	90	0.35	240	1501486	1502578	4.18	4.19	2.42	2.41
3	50	1	240	2149670	2148578	3.23	3.22	2.12	2.12
4	90	1	240	1730395	1730796	5.33	5.34	2.47	2.48
5	50	0.68	235	2037161	2038807	2.45	2.47	2.08	2.09
6	90	0.68	235	1565069	1565223	4.84	4.84	2.51	2.52
7	50	0.68	245	2004970	2004816	2.83	2.83	2.04	2.05
8	90	0.68	245	1589253	1587607	4.62	4.6	2.44	2.43
9	70	0.35	235	1755465	1754220	2.9	2.89	2.21	2.21
10	70	1	235	1984765	1984211	4.02	4.02	2.34	2.33
11	70	0.35	245	1777250	1777804	2.98	2.99	2.14	2.15
12	70	1	245	1947774	1949019	4.04	4.05	2.26	2.26
13	70	0.68	240	1804782	1804782	3.54	3.54	2.01	2.01
14	70	0.68	240	1804782	1804782	3.54	3.54	2.02	2.01
15	70	0.68	240	1804782	1804782	3.54	3.54	2.01	2.01
16	70	0.68	240	1804782	1804782	3.54	3.54	2.01	2.01
17	70	0.68	240	1804782	1804782	3.54	3.54	2.01	2.01

**Table 3:** Statistical ANOVA results for the responses (*Y*_1_ = area; *Y*_2_ = retention time; *Y*_3_ = tailing factor).

Result (ANOVA)	Area	Retention time	Tailing factor
Regression			
Sum of squares	23701247441	1.56	2.58
Degrees of freedom (df)	9	9	9
Mean squares	2633471938	0.17	0.28
*F* value	253.39	25.40	199.28
*P*	<0.0001	0.0002	<0.0001
Lack of fit tests			
Sum of squares	55661902.75	0.01	0.004
Degrees of freedom (df)	3	3	3
Mean squares	18553967.58	0.002	0.002
*F* value	4.34	0.16	0.91
*P*	0.10	0.92	0.51
Residual			
Sum of squares	72750482.75	0.05	0.01
Degrees of freedom (df)	7	7	7
Mean squares	10392926.11	0.01	0.001

**Table 4:** Linearity data of ATS.

Parameter	Observed result
Linearity range	5–30 *μ*g/ml
Calibration equation	*Y* = 98881*x* + 71.42
Slop	98881
Intercept	71.42
Limit of detection	0.254 *μ*g/ml
Limit of quantification	0.838 *μ*g/ml

**Table 5:** Precision and accuracy of different quality control samples of ATS.

Quality control sample	Concentration (*μ*g/ml)	Intraday precision and accuracy			Interday precision and accuracy		
		Experimental concentration	% RSD	Accuracy	Experimental concentration	% RSD	Accuracy
LQC	1.5	1.48 ± 0.04	2.70	98.66 ± 2.9	1.475 ± 0.04	2.71	98.33 ± 2.46
MQC	12.44	12.42 ± 0.3	2.41	99.84 ± 2.1	12.43 ± 0.3	2.41	98.91 ± 2.33
HQC	24	23.96 ± 0.43	1.79	99.83 ± 1.8	23.94 ± 0.3	1.25	99.75 ± 1.41

**Table 6:** Stability study of ATS at different quality controls.

Quality control sample	Refrigerated condition and time	Experimental concentration	% experimental drug	% RSD
LOC (1.5 *μ*g/ml)	Initial (0 h)	1.5 ± 0.02	100	1.33
	Short-term storage stability (25°C/24 h)	1.5 ± 0.03	100	1.33
	Long-term stability (−80°C, 30 days)	1.49 ± 0.03	99.33	2.01
	Three cycles freeze-thaw stability (three cycles, −20°C to 25 C)	1.48 ± 0.02	98.66	1.35
	Postpreparative stability (10°C, 24 h), initial (0 h)	1.49 ± 0.03	99.33	2.01

HQC (24 *μ*g/ml)	Initial (0 h)	24 ± 0.2	100	0.83
	Short-term stability (25°C, 24 h)	23.99 ± 0.3	99.95	1.25
	Long-term stability (−80°C, 30 days)	23.90 ± 0.3	99.58	1.26
	Three cycles freeze-thaw stability (three cycles, −20°C to 25°C)	23.92 ± 0.3	99.66	1.25
	Postpreparative stability (10°C, 24 h)	23.98 ± 0.3	99.91	1.25

## Data Availability

The data used to support the findings of this study are included within the article.
